# Editorial: Cellular heterogeneity in physiological and pathological myogenesis

**DOI:** 10.3389/fcell.2023.1235520

**Published:** 2023-06-15

**Authors:** Monica Dentice, Stefano Biressi, Lorenzo Giordani, Ombretta Guardiola

**Affiliations:** ^1^ Department of Clinical Medicine and Surgery, University of Naples Federico II, Naples, Italy; ^2^ Department of Cellular, Computational and Integrative Biology (CIBIO), University of Trento, Trento, Italy; ^3^ Centre de Recherche en Myologie, INSERM UMRS 974, Association Institut de Myologie, Sorbonne Université, Paris, France; ^4^ Stem Cell Fate Laboratory, Institute of Genetics and Biophysics “A. Buzzati-Traverso”, CNR, Naples, Italy

**Keywords:** heterogeneity, muscle stem cells, muscle regeneration, muscle aging, muscle disease

The skeletal muscle stem cell (MuSC) population has functional capacities to adapt to changing conditions throughout the lifespan, ensuring muscle maintenance, repair, and regeneration. MuSCs exhibit functional heterogeneity with distinct cellular subtypes fluctuating between phenotypic and functional states. This cellular plasticity enables MuSCs to respond to the varying requirements during skeletal muscle homeostasis, regeneration, aging, and diseases. The dynamic heterogeneity of the MuSC population determines the fate asymmetry, balancing self-renewal *versus* differentiation. Understanding genetic and non-genetic factors that underlie and shape MuSC flexibility is critical to comprehending their behavior in physiological and pathological contexts. Elucidating the molecular mechanisms and fundamental aspects of stem cell heterogeneity poses a challenge for regenerative medicine approaches aiming to therapeutically manipulate this promising cellular tool. The studies presented in this Research Topic, including one review and three original articles, provide insights into MuSC heterogeneity with a focus on their interactions with other cell populations and the extracellular matrix in skeletal muscle repair and disease, as well on their behavior in different muscle-related contexts, including the pathological setting. Wang and Zhou review the critical role of macrophages in skeletal muscle injury and repair. They discuss the heterogeneity of macrophage subtypes and their interactions with myogenic cells in homeostatic, regenerating, and dystrophic skeletal muscles. The authors provide insights into the cellular and molecular processes that regulate this interplay and the potential therapeutic manipulation of macrophages to promote injury repair. Andre et al. investigate the MuSC heterogeneity in response to inflammatory signals. In particular they focus on their capacity to produce chemokines and cytokines that act on the myeloid and myogenic cells during skeletal muscle regeneration. Using single-cell RNA sequencing, they identified cell clusters representing a continuum from activation to differentiation. Treatment with lipopolysaccharide (LPS) revealed a heterogeneous pattern of chemokines and cytokines expression. Among the cell clusters, the authors identified a previously unrecognized subset of MuSCs that may act as sensors for muscle infection or injury using the antiviral interferon pathway. Overall, the study underscores the importance of understanding the role of MuSCs in proinflammatory signaling during muscle regeneration and highlights the cellular plasticity of MuSCs as a key factor in maintaining muscle function throughout life. Muñoz et al. investigated the impact of matrix metalloproteinase-10 (MMP-10) on MuSCs aging and function, highlighting the importance of MMP-10 in maintaining healthy MuSCs and preventing premature aging. They demonstrated that loss of MMP-10 in young mice altered the composition of the muscle extracellular matrix (ECM) and disrupted the MuSCs’ niche, causing premature features of aging, such as reduced proliferation and differentiation capacity, and contributing to their functional decline. This study also showed that targeting MMP-10 could be a promising treatment for preventing muscle aging, as MMP-10 treatment rescued dystrophic MuSCs from cellular damage. Overall, this work provides relevant information on the aging of muscle stem cells and their ability to adapt during homeostasis, regeneration, and resistance to unfavorable conditions. Zhang et al. investigated the pathological mechanisms underlying oculopharyngeal muscular dystrophy (OPMD) in a knock-in mouse model of this disease (Pabpn1+/A17 mice). OPMD is known to be caused by mutations in the gene encoding the nuclear polyadenosine RNA binding protein PABPN1. The authors showed specific functional defects in the pharynx muscle and MuSCs derived from the pharyngeal muscle. They found that basal autophagy, which is crucial for normal MuSCs function, is higher in pharynx-derived myoblasts than in myoblasts derived from limb muscles. This observation suggests that muscle-specific cell-intrinsic differences in MuSCs function may contribute to OPMD.

